# Editorial

**Published:** 1849-02

**Authors:** 


### THE MEDICAL EXAMINER.

PHILADELPHIA, FEBRUARY, 1849.

### CHOLERA.

In the last number of the Examiner we presented our readers with a brief account of the progress which the Asiatic Cholera had made in the United States. In New York, where the disease first made its appearance, it has entirely disappeared within the last two weeks, having shown but little disposition to spread from the Quarantine Hospital, into which it had been introduced by some emigrants from France. How these persons had contracted the disease it is difficult to say, for, in a report made by Dr. Whiting, Health Officer at Quarantine, to the Sanatory Committee of the Board of Health, we learn that "all the persons who have been attacked, from the first case on board ship to the last, excepting the inmates of the hospital, *have been among two hundred and seventy Germans*, who have been living in Havre and its environs, where there has not been a single case of Cholera." Why these Germans were attacked with the disease, to the exclusion of the other passengers on board the ship New York, we cannot say, since, when they came on board, they seemed healthy and robust—had not been exposed to the cholera at home, and during the voyage we are assured by Dr. Whiting they "shared equally with the exempt the comforts and privations of a sea voyage, variations of wind and weather, have breathed the same air, and fed on the same food." This account presents to the medical mind two questions of importance and difficulty: 1st. How did the passengers of the ship New York become infected with cholera? 2d. How happened it that this disease only attacked persons from among two hundred and seventy Germans, to the exclusion of every one else? At present we do not propose to undertake the difficult task of replying to these interrogatories, but shall proceed with a brief detail of the progress of cholera in our country. Of its progress in New York nothing more need be said, since the disease has been chiefly confined to the Quarantine—having only appeared in the city in one or two instances.

From the report of Dr. Whiting, we find that most of the persons attacked with cholera presented all or most of the following symptoms: "Vomiting and purging of thin discharges, sometimes at first light brown, but generally from the first of a white or yellowish-white or pearl colour, with white flocculi, forming a thicker whitish sediment on standing a short time. They are well described as *rice water* evacuations. In some cases a half gallon has been vomited, but generally in smaller quantities. A child six years old vomited fully this quantity at once, had no other evacuation, and afterwards recovered. The vomiting is usually accompanied by great uneasiness and pain, particularly at the epigastrium. In some cases vomiting has existed without purging, and vice versa. In several cases neither vomiting nor purging, but the stomach and bowels were found filled after death with the same fluid. One or more large worms, the lumbrici, have been discharged, either by vomiting or the bowels, in a large majority of cases. This fact has been before remarked. The tongue and breath are icy cold; sometimes the tongue is clean, but generally slightly coated. The voice becomes weak and husky, and with a great effort the patient speaks in a thick whisper. The skin assumes a livid colour, becomes cold and clammy, and when pinched up remains so for a short time. The countenance wears a haggard, sunken look; the eye is dull and heavy, although the pupil is somewhat dilated. The extremities are shrivelled, the fingers and toes and nails resembling those that have been long in the water, and of a purple hue. All these cases have been affected with cramps and spasms of the extremities and abdomen; in some a slight but generally a very painful symptom. The pulse, from the inception of the real attack, becomes small and frequent, from 110 to 140, according to the progress of the disease, and, in the stage of the collapse, entirely lost at the wrist for hours. The breathing laboured and hurried, and in cases where the spasms were severe, occasionally suspended momentarily." In the report we find it stated that diarrhœa preceded an attack of cholera only in a few instances; indeed, there seems to have been great difficulty in detecting any other stage of the disease than that of collapse, notwithstanding every effort was made, by repeated visits, &c., to ascertain the earliest deviations from health. Dr. Whiting says, "the number or *apparent* violence of the symptoms formed no criterion for the prognosis. Fatal results followed, in a number of cases, in a few hours, where the dejections were slight, and spasms and other violent symptoms were absent."

The post-mortem appearances consisted in engorgement of all the internal organs, "either from actual congestion or the retention of the cruor of the blood, while the serum is drained off." No alteration in structure was observed in any case. The skin was shrunk and shrivelled, and of a livid colour. The bladder was contracted, and in most of the cases there was an entire suppression of urine.

The average duration of the disease, up to the time of the report, was about ten hours—in children it was not more than *four*.

Upon the landing of these infected persons at the Quarantine, it appears that six others, inmates of the Marine Hospital, became affected in a similar way, though they had been only two days in proximity with the emigrants of the ship "New York." One person, being discharged, went up to the city, and though he had only remained *a little over a day* in the same enclosure with the cholera patients, was attacked with the disease and died. Dr. Whiting adds, that "on perceiving this communication of the disease to the convalescents, I immediately sent them away, and distributed them through the other hospitals, since which three others have been attacked, two of whom have died, but none other (than those at first exposed at the public stores) have been affected. These had all been inmates of the hospital for weeks, were ready to be discharged, and had but a limited exposure for forty-eight hours to the influence of the disease."

When the disease first broke out on board the ship New York, "the ship was in N. Lat. 42°, Long. 61, about 140 miles S. S. W. from Sable Island. On the 23d and 24th, the two days preceding the appearance of the cholera, the wind was N. N. W. On the 25th it changed to the southward, with squalls and rain. In the morning the barometer was at 30 inches, and fell during the day to 29½ inches; thermometer 60°, Fahrenheit. Sunday and Monday, 26th and 27th, wind westerly and fresh. Tuesday, 28th, moderate from N. W.; barometer 30; thermometer 42°."

Dr. Whiting next proceeds to notice the treatment adopted in thirty cases, which had occurred at the Quarantine up to the 11th of December. Of these thirty, twenty were fatal. Of the external treatment he says, the most efficient mode of restoring the heat of the surface consisted in enveloping the patient in warm blankets—in the application of mustard to the stomach and bowels, and to the upper and lower extremities—in friction with hot tincture of capsicum, and the conveyance, by means of suitable apparatus, of a constant stream of hot air under the bed clothes.

Various plans of internal treatment were tried. Mustard emetics, without bloodletting, proved successful in only two cases. Unless reaction took place after emesis, the irritation of the stomach and the prostration seemed very much increased. In eight cases, large doses of calomel, capsicum and camphor, in conjunction with external frictions, produced so little good effect, as not to encourage a continuation of the treatment. Chloroform was used, "and at first gave some hope that it would prove a successful remedy; but no other permanent good has resulted from its use but to relieve the spasms and cramps." For this purpose it is an invaluable remedy in the disease. The saline mixture failed to produce any good effect, owing, probably, to the fact that in those cases in which it was administered the power of absorption was destroyed. Acetate of lead and opium were administered in large and repeated doses, but without any good effect. "The treatment," says Dr. Whiting, "that has proved of most service, has been calomel in scruple doses, combined with opium and camphor, followed, at two or three hours' interval, by smaller doses of calomel, until reaction is indicated by some action of the liver. This plan, combined with the faithful application of external heat, &c., I am satisfied, has proved of most advantage in the cases that have come under my notice. Every case in which the slightest bilious evacuation has been procured, has commenced to recover from that moment, and although of itself unable to effect the reaction necessary for its own peculiar action, calomel will doubtless always prove the most potent auxiliary in the catalogue of remedies for cholera."

Subsequent to the 11th of December, which is the date of the preceding account, Dr. Whiting states that he has, up to December 19th, met with thirty-three new cases—all of which were from the same class of German emigrants, except three—one a Frenchman from on board the ship New York, the other two inmates of the hospital previous to the arrival of the infected vessel. Of these thirty-three cases, which were treated with calomel, opium and camphor, only nine have died; and "as there appeared to be no difference in the severity of the symptoms at the outset of the disease, he cannot but attribute the diminished fatality to a more happy plan of treatment." When the disease is yielding to these remedies, we observe a subsidence in the pain and spasms, with warmth of skin and restoration of pulse. The quantity and frequency of the discharges diminish, and their appearance changes from the thin rice water, to a greenish and thin brown or brownish-yellow colour.

The cholera has also made its appearance in the southern section of the United States. It is supposed to have been introduced into New Orleans by passengers arriving from Havre on board the ship Swanton. They were landed the 11th of December, and on the 12th the first cases made their appearance. Since then the following statement, said to be official, in regard to the number of persons who have died of cholera, has been received:

| | Asiatic Cholera. | Cholera. | Total. |
|---|---|---|---|
| Week ending December 16, - - | 3 | 14 | 17 |
| December 16 to 21, - - - - - | 53 | 34 | 87 |
| December 22, - - - - - - | 39 | 7 | 46 |
| December 23, - - - - - - | 56 | 15 | 71 |
| December 24, - - - - - - | 76 | 8 | 84 |
| December 25, - - - - - - | 69 | 8 | 77 |
| December 26, - - - - - - | 8 | 46 | 54 |
| December 27, - - - - - - | 22 | 39 | 61 |
| December 28, - - - - - - | 4 | 88 | 92 |
| December 29, - - - - - - | 84 | | |
| December 30, - - - - - - | 77 | | |
| December 31, - - - - - - | 71 | | |
| January 1, - - - - - - | 67 | | |
| January 2, - - - - - - | 84 | | |
| January 3, - - - - - - | 67 | | |
| January 4, - - - - - - | 39 | | |
| January 5, - - - - - - | 44 | | |

Whole number of deaths from Cholera, 1,115; reported Asiatic, 870; Cholera otherwise designated, 245.

The disease is said to be confined principally to foreigners, not showing itself to any great extent among the natives of New Orleans, or those resident there from other States. In a letter to the Editor of the Boston Medical and Surgical Journal, from Dr. Wederstrandt, of the Charity Hospital, New Orleans, we extract the following:

"On the 12th of the present month the cholera broke out in this hospital. The two first cases were a man and a woman, who were brought, in the last stage of the disease, from the ship Swanton, which had just arrived from Havre. This vessel left Havre with all the passengers and crew in good health, neither was the cholera in that port when she left; but some of the passengers were from a part of Germany where the cholera was raging. When at sea two weeks, the disease broke out on board, and 17 persons died in a few days, and were thrown overboard. At the time she reached here, but two were sick on board, and they were brought to this hospital. The very next day numerous cases appeared all over the city, but principally in the houses nearest to the shipping, or among persons employed on the wharves. Since the middle of this month [December] we have admitted between 40 and 50 persons with this disease every day; upwards of 50 cases have originated in the house among the convalescents of other diseases and the attendants; three of the washer-women have taken the disease, and two have died. The disease here seems to consist of three stages in most cases: first, a feeling of malaise and diarrhœa; next comes on the vomiting and purging of rice water discharges, and cramps; thirdly, the cold stage, with the clammy sweat and suppression of urine. The intelligence remains intact until very late. The disease has proved fatal here in so short a time as three hours. Oftener it is protracted to twelve and fifteen, and rarely beyond twenty-four hours. The violence of the pain in the stomach, and vomiting and purging, does not always afford a criterion for an unfavourable prognosis, for many patients recover rapidly in a few hours after being so attacked, declaring themselves nearly as well as ever. About half an hour after death, the body, which was as cold as ice just before, becomes as warm as in health; and the cramps or contractions of the muscles, which annoyed the patient so much during life, continue for at least half an hour, and in some cases nearly an hour after death.

During the first days of the epidemic, nearly all the cases proved fatal; but within the last few days it seems to be rather on the decline, as our admissions and deaths have decreased, and we begin to number many cures, or rather recoveries. We treat the disease on general principles, and according to the indications of each individual case. In the early stage, we have had reason to be satisfied with the preparations of opium and counter-irritants. Some physicians use a large dose of opium and quinine in the beginning, when they get their cases early; they give from thirty to forty drops, to a drachm of tincture of opium, with half a drachm of quinine, for a single dose, and speak highly of their success. In a few cases I have thought that the practice did good, but I have not used it to any great extent. When brought to us, which they generally are, in the cold stage, we use stimulants externally and internally, with nourishing broths, and several have re-acted under this treatment, and finally recovered. Males and females, young and old, are alike subjects for this disease; but far more men than women are attacked. We have seen many children die of it, some under five years, and a few old people at a very advanced age. Dr. Watson, in his very interesting and valuable Lectures on the Practice of Medicine, has given a most correct description of the disease as it now prevails among us, and I believe it to be identical with the Asiatic cholera, which he so ably describes."

From New Orleans it has spread to other places along the borders of the Mississippi and its tributaries. In Texas it has also made its appearance, in a form as aggravated as that which marked the disease in New Orleans. At Lavaca, Texas, the 8th U. S. Infantry has been severely dealt with. The last account from these different places indicate that the disease is, for the time at least, abating. Of the treatment adopted in the management of this disease, in the places above alluded to, we are not informed, as our exchange Journals have not as yet come to hand.

---

DEATH OF MR. SAMUEL COOPER.

Within a year English Surgery has lost two of its brightest ornaments, *Robert Liston and Samuel Cooper.*

By a singular coincidence, Mr. Cooper's death took place upon the anniversary of that of his friend and colleague, Mr. Liston, on the 9th of December, 1848.

The name of Samuel Cooper is as familiar as "household words," to every medical man on this side of the Atlantic, as the author of the "Surgical Dictionary," and "First lines of Surgery." In early life he was a member of the medical staff of the army, and was present in the memorable Walcheren expedition. In his later years he filled several highly responsible and important public offices. As a surgeon he was much distinguished, and as a teacher eminently successful, having for seventeen years filled the chair of Surgery in University College. He was also surgeon and consulting surgeon to the University Hospital, from the time of its foundation, till his withdrawal from both, in April, 1848, which separation was caused by a misunderstanding with some of his colleagues. A very general impression exists in his own country, that the irritation and mortification resulting from this misunderstanding, may have hastened his death, the immediate cause of which is stated to have been suppressed, or retrocedent gout.

"Professor Samuel Cooper, well known all over the world for the variety and extent of his surgical information, as exemplified in that almost unequalled production, the 'Dictionary of Surgery,'"* was, at the time of his decease, in the 68th year of his age.

---

We are reminded that in this country, we too, in the brief space of two years, have been called upon to mourn the voids created by the hand of death, in our own circle. The memory of Hewson, McClellan, and Randolph, of this city, and Warner, of Richmond, is still fresh in our hearts; and though we look in vain for them in their familiar places, their examples and their teachings are still left to us as a legacy that gold cannot value.

*Sketches of eminent British Surgeons, by Wm. Gibson, M. D., Professor of Surgery in the University of Pennsylvania.

AMERICAN MEDICAL ASSOCIATION.

By desire of the Committee of Arrangements, the undersigned request all societies and other institutions authorized to appoint delegates, to send correct lists of those chosen to attend the *next annual meeting*, to Dr. Henry J. Bowditch, Boston, on or before the 1st of April, 1849.

Editors of Medical Journals are respectfully solicited to circulate the above request, a compliance with which will greatly facilitate the organization of the Delegates when assembled.

Alfred Stillé,
Henry J. Bowditch,
Secretaries of A. M. A.

---

THE SOUTHERN MEDICAL AND SURGICAL JOURNAL.

We regret to learn from the last number of this Journal, that there is a prospect of its discontinuance, from want of adequate support. We sincerely hope, for the sake of the profession in the South and West, that this will not be allowed to occur, but that they will rally to the aid of the "oldest, the most prompt, and the only monthly medical publication" of that section.

---

At the first Stated Meeting of the Philadelphia County Medical Society, held January 16th, 1849, the following officers for the current year were elected.

*President*,—Samuel Jackson, M. D., (late of Northumberland.)
*Vice Presidents*,—George Fox, M. D., T. F. Betton, M. D.
*Recording Secretary*,—D. Francis Condie, M. D.
*Corresponding Secretary*,—Henry S. Patterson, M. D.
*Treasurer*,—M. M. Reeve, M. D.
*Censors*,—Thomas Hobson, M. D., Wilson Jewell, M. D., J. Forsyth Meigs, M. D., Isaac Parrish, M. D., David H. Tucker, M. D.

At the adjourned meeting held on Tuesday afternoon, January 30th, at 3½ o'clock, delegates were to be elected to the State Society, and to the National Medical Association, previously to which time, those gentlemen whose names were received at the meeting held December 18th, 1848, as constituent members, were requested to call on the Secretary and sign the constitution, which is essential to entitle them to full membership.

[We have not heard the result of this adjourned meeting, but will report the proceedings in our next number.—Eds.]

